# Development of a Homogeneous Trastuzumab-Triptolide Conjugate for Targeted Therapy of HER2-Overexpressing Ovarian Cancer

**DOI:** 10.1021/acs.bioconjchem.6c00171

**Published:** 2026-08-21

**Authors:** Idowu E. Fadayomi, Maria Jangan, Dilna Varghese, Elisabete Pires, James McCullagh, Alan Richardson, Adrian Murray Brunt, Wen-Wu Li

**Affiliations:** † School of Life Sciences, 4212 Keele University, Staffordshire ST4 7QB, U.K.; ‡ School of Health Allied Professional and Pharmacy, 158984 Keele University, Staffordshire ST5 5BG, U.K.; § Department of Chemistry, 6396 University of Oxford, Oxford OX1 3TA, U.K.; ∥ School of Medicine, Keele University, Staffordshire ST5 5BG, U.K.

## Abstract

Ovarian cancer remains a lethal malignancy due to chemoresistance and toxicity, which limits the dose of chemotherapy that can be used, necessitating the development of more targeted therapies such as antibody-drug conjugates (ADCs). However, conventional ADCs suffer from heterogeneity. This study aimed to develop a stable, homogeneous ADC by utilizing a bifunctional dibromomaleimide (DBM) linker to cross-link antibody cysteine residues via disulfide-bridging. A DBM linker was synthesized from 3,4-dibromofuran-2,5-dione and bound to the cytotoxic agent triptolide. This triptolide payload was then conjugated to trastuzumab via site-specific disulfide rebridging to yield a homogeneous trastuzumab-triptolide conjugate to target human epithelial growth factor receptor 2 (HER2) on ovarian cancer cells. The study evaluated the ADC’s efficacy against SKOV-3 (high HER2 expression) and OVCAR-8 (low HER2 expression) cell lines. The results showed that the ADC was slightly more potent in SKOV-3 cells, yielding lower IC_50_ values compared to OVCAR-8. Mechanistic studies of the ADC via flow cytometry revealed that the ADC induced significant apoptosis and cell cycle arrest, characterized by a concentration-dependent increase in Caspase-3/7 expression and distinct alterations in cell population distribution. Furthermore, ADC treatment led to a concentration-dependent decrease in HER2 levels in SKOV-3 cells, confirming successful targeting. The study demonstrates that converting conventional maleimides into bifunctional DBM linkers allows the production of a homogeneous ADC via disulfide-bridging. This approach offers a promising strategy for developing potent anticancer therapeutics with improved selectivity for HER2-overexpressing ovarian cancers.

## Graphical abstract

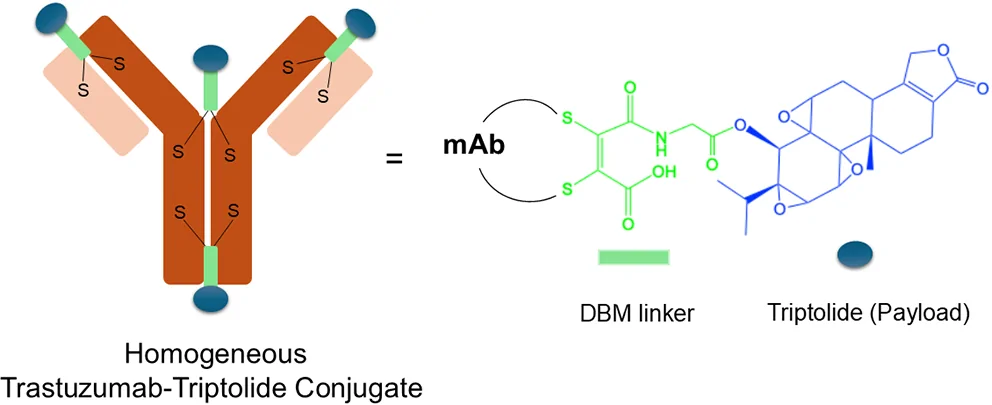

## 1 Introduction

Ovarian cancer is the sixth most common cause of female cancer in the UK and remains the deadliest cause of gynecological malignancy.^1^ Its high mortality rates can be attributed to its late presentation in women, meaning the disease is often detected in the advanced stages. At this point, it has effectively become metastatic within a large proportion of the abdomen.^2,3^ The low early detection rate or preventative methods for epithelial ovarian cancer (EOC), the most common form, are in large part due to the lack of symptoms in early-stage disease.^4^ A widely accepted pathogenesis for EOC is yet to be established, and the disease is often characterized by gene amplification and whole genome duplication, rather than activation of oncogenes through smaller mutational events.^5^ While treatment with paclitaxel and carboplatin or cisplatin chemotherapy has successfully allowed some women to show a complete response, most patients in the late stage will develop a recurrence of their disease. As is the impediment with many other cancers, EOCs may become chemoresistant. A further limiting factor is the significant toxicity resulting from the need for repeated chemotherapy.^2^ The recent introduction of PARP inhibitors has been transformative in patients with defects in homologous recombination (HR), but it is less effective in patients with normal HR function.^6^ Furthermore, current cancer therapies, including chemotherapy, radiotherapy, antibody immunotherapy, and targeted therapies, are also limited by toxicity and poor efficacy.^7^ There is thus a great clinical need for the development of more sophisticated, safer, and efficacious anticancer treatments.

Human epithelial growth factor receptor 2 (HER2) is well-known to play an important role in the development of ovarian and breast cancer and has been clinically utilized as a target for antibody and antibody-drug conjugate (ADC)-based therapy.^8,9^ It is also highly expressed in a subset of ovarian cancers, although the expression varies between different histological subtypes, with the most significant expression being observed in mucinous ovarian cancers.^10,11^ ADCs represent a rapidly evolving class of potent, targeted therapeutics designed to address the limitations of conventional chemotherapy. By combining the exquisite specificity of antibodies with highly cytotoxic payloads, these constructs offer a promising strategy to overcome the current scarcity of effective, low-toxicity treatments.^12,13^ ADCs comprise of a specific monoclonal antibody and a linker with a cytotoxic payload (or warhead) as a tripartite drug. As of 2025, 15 ADCs have been approved by the FDA for the treatment of hematologic and solid malignancies.^14^ The first FDA-approved anti-HER2 antibody, trastuzumab, has served as a foundational scaffold for several ADCs, including the clinically approved ado-trastuzumab emtansine (T-DM1) and trastuzumab deruxtecan (DS-8201).^13^ Elahere (mirvetuximab soravtansine) is the first FDA-approved ADC for FRα-positive, platinum-resistant ovarian cancer, showing improved response rates over standard chemotherapy.^15^ Despite these developments, the current generation of ADCs is not without problems, largely undesirable pharmacokinetics due to heterogeneity caused by nonspecific conjugation, resistance mechanisms, systemic toxicities, and a limited number of warheads being in current use.^14,16–18^ To overcome these shortcomings, novel methods for site-specific conjugation and expanding the range of payloads with high potency are needed.^18,19^ Recently, disulfide-bridging linkers using a bis-reactive linker moiety have been effectively employed to enable reaction with reduced cysteines to reform a bridge, leading to site-specific, covalent rebridging of the antibody,^20–23^ retaining structure and antigen-binding capability without the need for complex genetic engineering of antibody. To expand the cytotoxic library for novel ADC generation against ovarian cancer, triptolide is an excellent candidate. Triptolide is a natural diterpenoid isolated from the medicinal plant Tripterygium wilfordii Hook. It is most known as the Thunder God Vine or Lei Gong Teng,^24^ and targets a subunit of the transcription factor TFIIH and inhibits its DNA-dependent ATPase activity.^25^ It shows an entirely orthogonal mechanism of action from other antimitotic agents used in ADCs and so has the potential to target paclitaxel-resistant ovarian cancer.^26^ While various triptolide analogues have been developed as clinical candidates,^27^ the delivery of triptolide via site-specific antibody conjugation is anticipated to significantly enhance its therapeutic window. Recently, triptolide-based ADCs have been made to target CD25 antigen expressing in malignant mesothelioma and leukemia,^28^ EGFR (cetuximab-triptolide conjugate) in lung cancer,^29^ HER2 in breast cancer,^30^ CD19 in systemic lupus erythematosus,^31^ and PD-L1 in pancreatic cancer^32^ using various linker chemistries. A novel dual-payload ADC platform incorporating exatecan and triptolide^33^ has been developed to enhance antitumor efficacy and overcome resistance in tumors overexpressing TROP2 or HER3.

This study proposes newly found optimization strategies for linker chemistry using disulfide bridging, combined with the potency of triptolide and the highly targeted affinity of trastuzumab, which have the potential to support the development of a novel trastuzumab-triptolide conjugate (ADC).

## 2 Results

### 2.1 Synthesis of Triptolide with a Linker and Trastuzumab Triptolide Conjugate (ADC)

Triptolide with a DBM linker (TP-DBM) was synthesized by coupling 3,4-dibromomaleimide-*N*-acetic acid to the 14-hydroxy group of triptolide in the presence of *N,N*′-diisopropylcarbodiimide (DIC) and 4-dimethylaminopyridine (DMAP) (Figure 1A). The product was purified by semipreparative high-performance liquid chromatography (HPLC) and characterized by ^1^H and ^13^C NMR data (Table 1) and mass spectrometry (Supporting Information). This payload derivative was further utilized to conjugate with trastuzumab after reduction with the reducing agent tris­(2-carboxyethyl)­phosphine hydrochloride (TCEP) to produce a novel ADC via disulfide bond rebridging (Figure 1B-D). A single, symmetric ADC peak on HPLC indicates a homogeneous population with a consistent antibody–drug ratio (ADR) (Supporting Information). Matrix-assisted laser desorption/Ionization time-of-flight mass spectrometry (MALDI-TOF MS) data (Figure 2) showed the mass of the parent antibody trastuzumab is 148261 corresponding to its glycosylated form,^34^ the mass of the ADC was found to be 149982 corresponding to the site-specific conjugation of 4 molecules of triptolide to the four-disulfide bond positions via rebridging. Size exclusion chromatography (SEC) showed that the ADC is predominantly monomeric (87%), with a minor dimer fraction (13%), similar to the unmodified antibody. The 0.9 min decrease in retention time of the ADC compared to trastuzumab indicates an increase in molecular size, consistent with the MALDI-MS results (Supporting Information).

**Figure 1:**
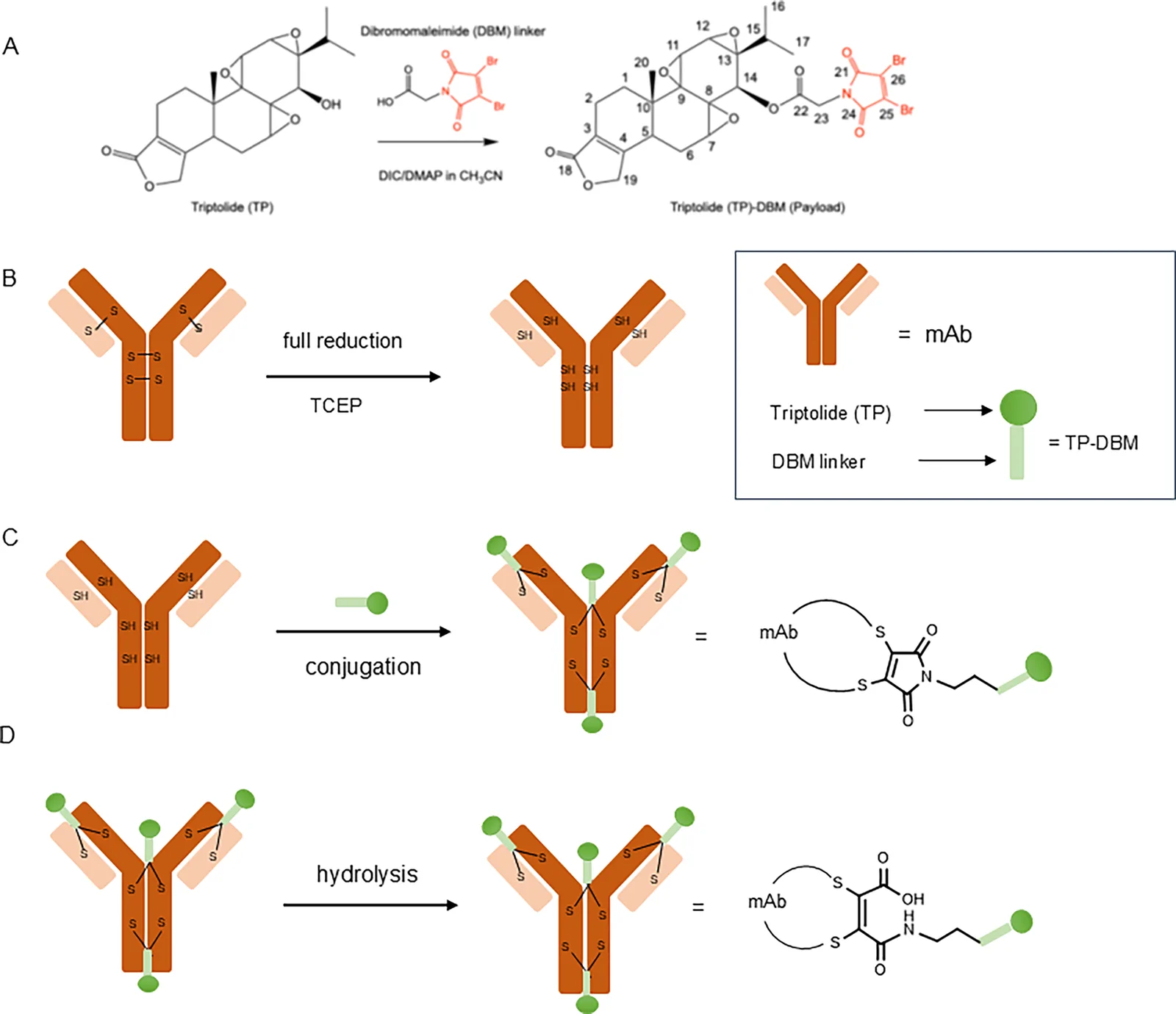
Scheme of synthesis of trastuzumab-triptolide conjugate (ADC). A) Synthesis of triptolide-dibromomaleimide payload (TP-DBM). It is made by coupling 3,4-dibromomaleimide-*N*-acetic acid to the 14-hydroxy group of triptolide via an ester bond formation in the presence of DIC and DMAP in acetonitrile at room temperature. B) Reduction of disulfide bonds of trastuzumab by TCEP. C) Reaction of the pair of free thiols with TP-DBM by replacement of two bromides to give trastuzumab-triptolide conjugate. D) The hydrolysis of the conjugate to yield a final stable form of ADC.

**Figure 2:**
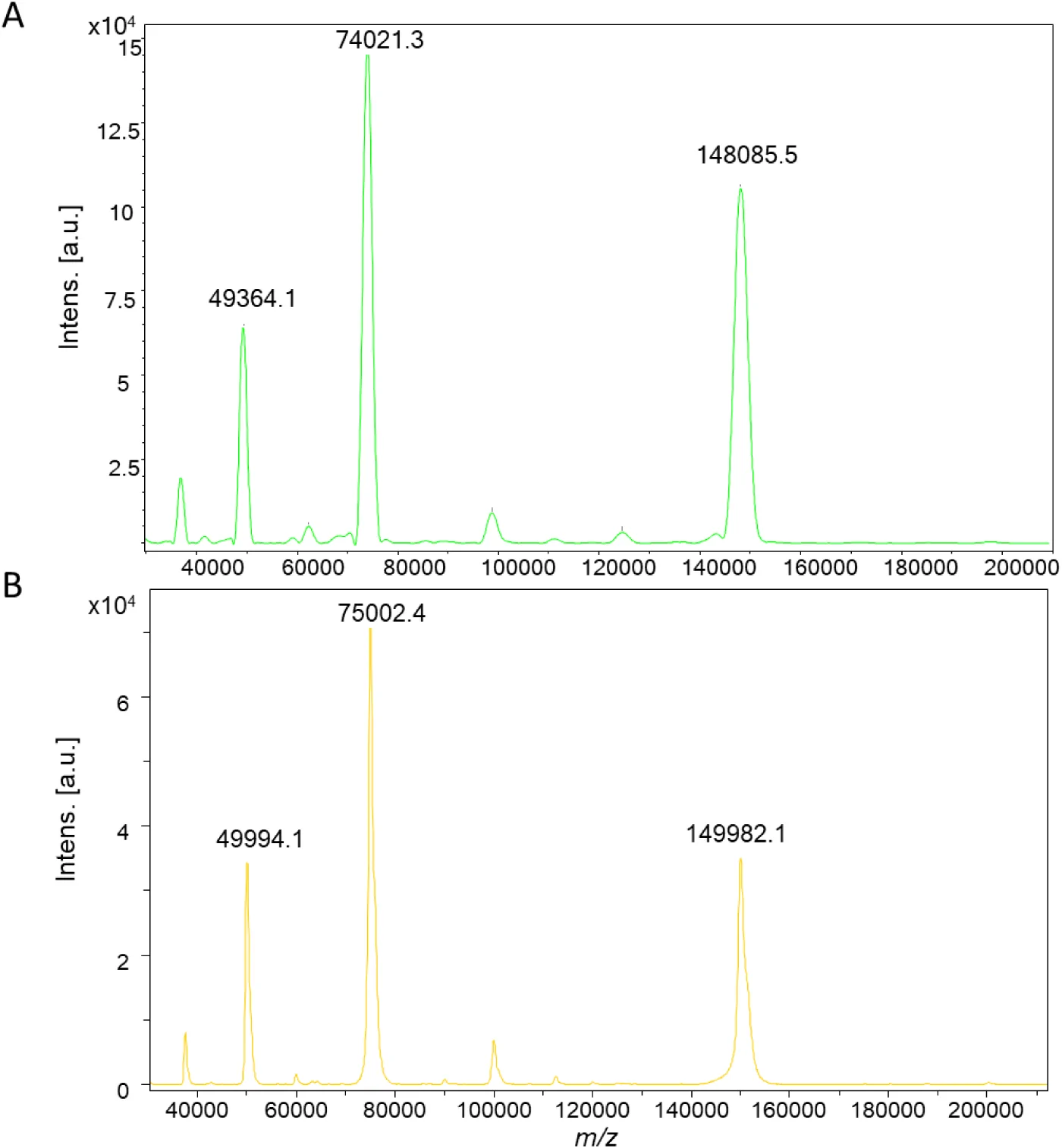
MALDI-TOF MS spectra of the unmodified trastuzumab (A) and trastuzumab-triptolide conjugate (ADC) (B). The mass difference (1896 Da) between 149982 for the ADC and 148086 for trastuzumab corresponds to approximately 4 molecules of TP-DBM linker.

**Table 1:** ^1^H NMR (400 MHz) and ^13^C NMR (100 M Hz) Chemical Shift Assignments of TP-DBM Payload in CDCl_3_

TP-DBM Payload		
Number	δ_*H*_	δ_*C*_
1	1.28 (1H, m), 1.58 (1H, dd, *J* = 4.0, 8.0 Hz)	29.83
	(1H, dd, *J* = 4.0, 8.0 Hz)	
2	1.86 (2H, m)	17.04
3	-	125.67
4	-	160.02
5	2.47 (1H, s)	40.36
6	1.84 (1H, m), 2.15 (1H, m)	23.39
7	3.43 (1H, d, *J* = 8.0 Hz)	59.43
8	-	61.15
9	-	62.96
10	-	35.65
11	3.82 (1H, d, *J* = 4.0 Hz)	55.28
12	3.53 (1H, d, *J* = 4.0 Hz)	55.08
13	-	63.51
14	5.02 (1H, brs)	73.38
15	2.32 (1H, m)	28.35
16	0.88 (3H, d, *J* = 8.0 Hz)	16.75
17	0.97 (3H, d, *J* = 8.0 Hz)	17.48
18	-	173.44
19	4.70 (2H, s)	70.07
20	1.07 (3H, s)	13.79
21	-	163.22
22	-	165.98
23	4.41 (1H, d, *J* = 20 Hz)	40.19
	4.57 (1H, d, *J* = 20 Hz)	
24	-	163.22
25	-	129.90
26	-	129.90

### 2.2 Antiproliferative Activity in Ovarian Cancer Cell Lines

The *in vitro* cytotoxic profiles of the ADC, trastuzumab, triptolide, and paclitaxel were evaluated against the SKOV-3 and OVCAR-8 ovarian cancer cell lines, with the corresponding IC_50_ values summarized in Table 2. The ADC exhibited significantly greater potency in inhibiting cell proliferation and survival compared with both unconjugated trastuzumab and paclitaxel across both cell lines. While the ADC demonstrated cytotoxic potency comparable to that of triptolide, the derivative TP-DBM showed a marginal reduction in potency against both SKOV-3 and OVCAR-8 relative to the parent triptolide compound.

**Table 2:** *In Vitro* Cytotoxicity After 72 H of Treatment with ADC, Trastuzumab, Triptolide, TP-DBM and Paclitaxel

	IC_50_ (mean ± SEM) (nM) (*n* = 3)	
Compounds	SKOV-3	OVCAR-8
ADC	5.0 ± 0.15	9.0 ± 0.38
Trastuzumab	>17.0	>17.0
Triptolide	3.6 ± 1.1	18.2 ± 8.7
TP-DBM	13.2 ± 2.5	20.2 ± 3.3
Paclitaxel	29.7 ± 11.9	40.3 ± 5.4

The levels of HER2 shedding from the cell surface^35^ in SKOV-3 and OVCAR-8 were quantified in the culture media by enzyme-linked immunosorbent assay (ELISA). The results indicated that SKOV-3 had a significantly higher HER2 level than OVCAR-8 (Figure 3A). SKOV-3 presented significantly lower HER2 expression after treatment with ADCs and trastuzumab (Figure 3B).

**Figure 3:**
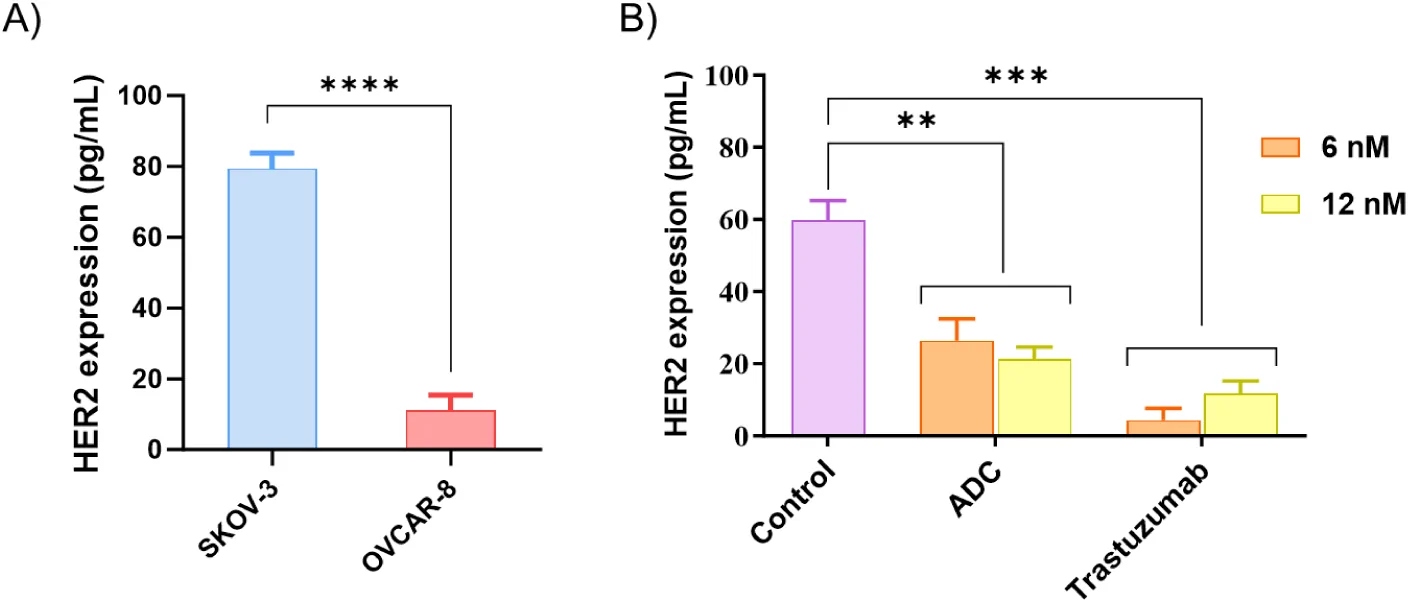
Comparison of the shed HER2 levels by ELISA assay. (A) SKOV-3 showed a higher HER2 concentration than OVCAR-8 cells (*p* < 0.0001). (B) HER2 concentrations for SKOV-3 drops significantly after treatment with ADC at 6 or 12 nM (*p* = 0.0034, control vs ADC); *p* = 0.0002 (control vs trastuzumab); Trastuzumab served as the positive control. Untreated SKOV-3 cells served as the negative control. The significance levels of *p* < 0.01, *p* < 0.001, and *p* < 0.0001 are represented **, ***, and ****, respectively. Three repeats were performed.

### 2.3 Apoptotic Activity in SKOV-3 Cells

To further investigate the mechanism of action of ADC, all subsequent experiments were done using only SKOV-3 cells due to its higher expression of HER2 than that of OVCAR-8 cells. The WST1 assay (Table 2) measures only the metabolic activity of a cell population, so cell morphology, caspase 3/7, and flow cytometry assays were all performed to investigate the apoptotic activity of the ADC in SKOV-3 cells. Morphological changes such as cell shrinkage, plasma membrane blebbing, and cell detachment (Figure 4), all indicative of apoptosis, were observed 48 h post-treatment with 6 nM ADC and became more pronounced 72 h post-treatment with ADC. Treating SKOV-3 cells with paclitaxel showed similar time-dependent apoptotic activity to that of the ADC. Triptolide treatment showed the most significant morphological changes, whereas treatment with trastuzumab showed little cell shrinkage and cell detachment.

**Figure 4:**
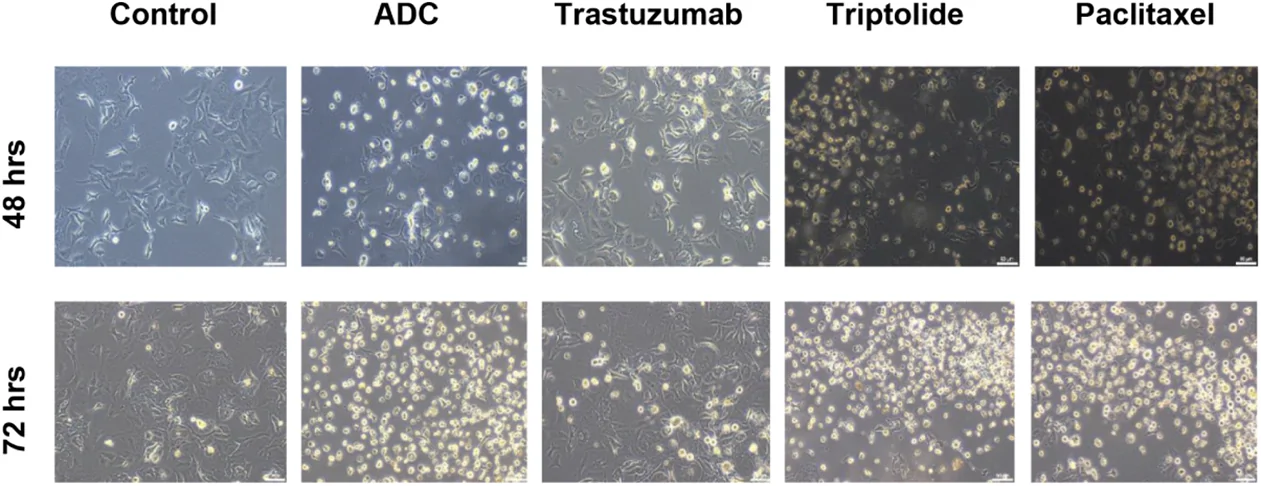
Morphological features of SKOV-3 after 48 and 72 h treatment with ADC and trastuzumab at 6 nM, and triptolide and paclitaxel at 10 nM. Untreated cells served as the control.

The caspase-3/7 activity in SKOV-3 was higher after ADC treatment and increased significantly with higher concentrations of the ADC used (Figure 5). Likewise, triptolide and paclitaxel exhibited significant caspase-3/7 activity, with a dose-dependent relationship. No significant caspase-3/7 activity was observed following the addition of trastuzumab.

**Figure 5:**
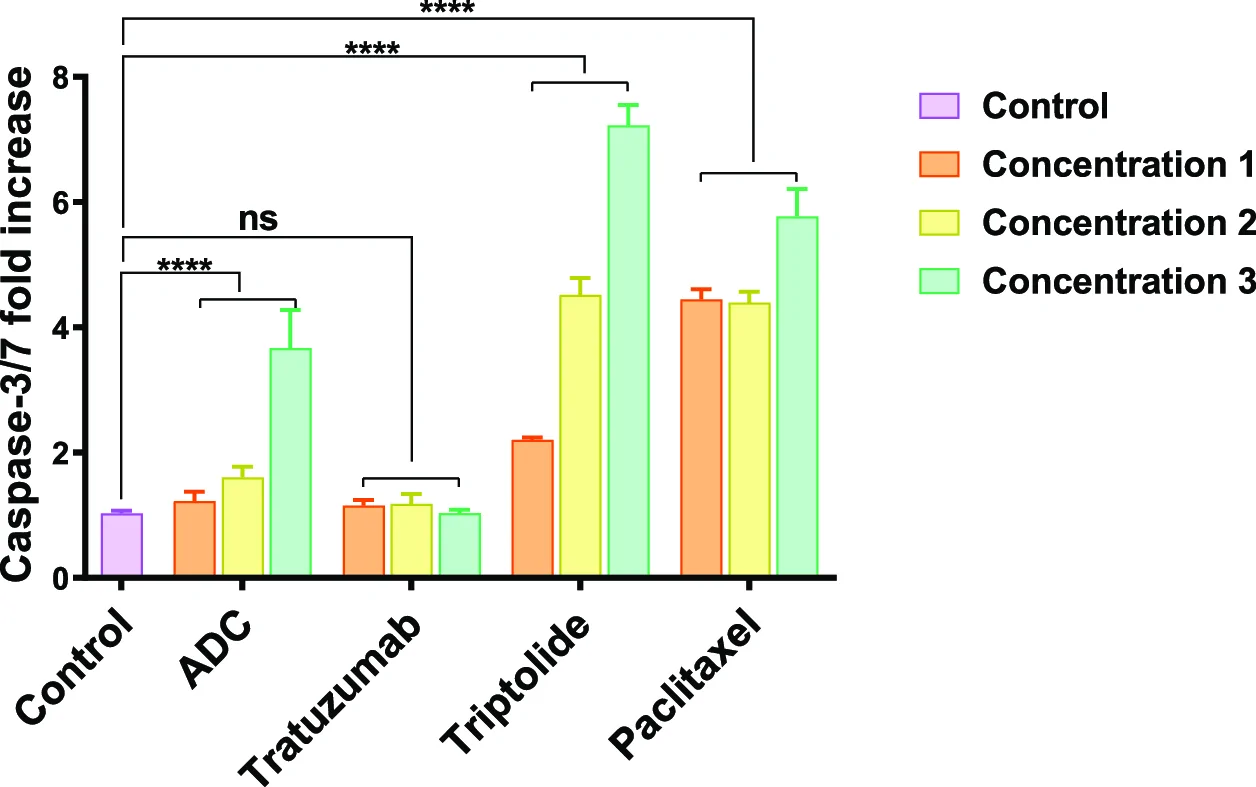
Caspase-3/7-fold increase for SKOV-3 after treatments ADC, trastuzumab, triptolide and paclitaxel. ADC and trastuzumab were used at 6, 12, and 24 nM. Meanwhile, triptolide and paclitaxel were used at 10, 20, and 40 nM. A significance level between control and treatment of *p* < 0.0001 is represented by **** whereas ns represents no significant difference.

Flow cytometric analysis of SKOV-3 cells treated with 6 nM ADC revealed a total apoptotic population of 34% (comprising 6% early and 28% late apoptotic cells), representing a significant increase relative to the untreated control sample. Treatment with higher concentrations of the ADC (12 and 24 nM) further augmented this effect, demonstrating a clear dose-dependent proapoptotic response. In comparison, unconjugated trastuzumab induced only 24% apoptosis at 24 nM. Triptolide showed around 30% apoptotic activity at concentrations of 10 nM. Paclitaxel exhibited a similar dose-dependent trend but with lower overall potency, yielding 15%, 21%, and 34% total apoptosis at 10, 20, and 40 nM, respectively (Figure 6).

**Figure 6:**
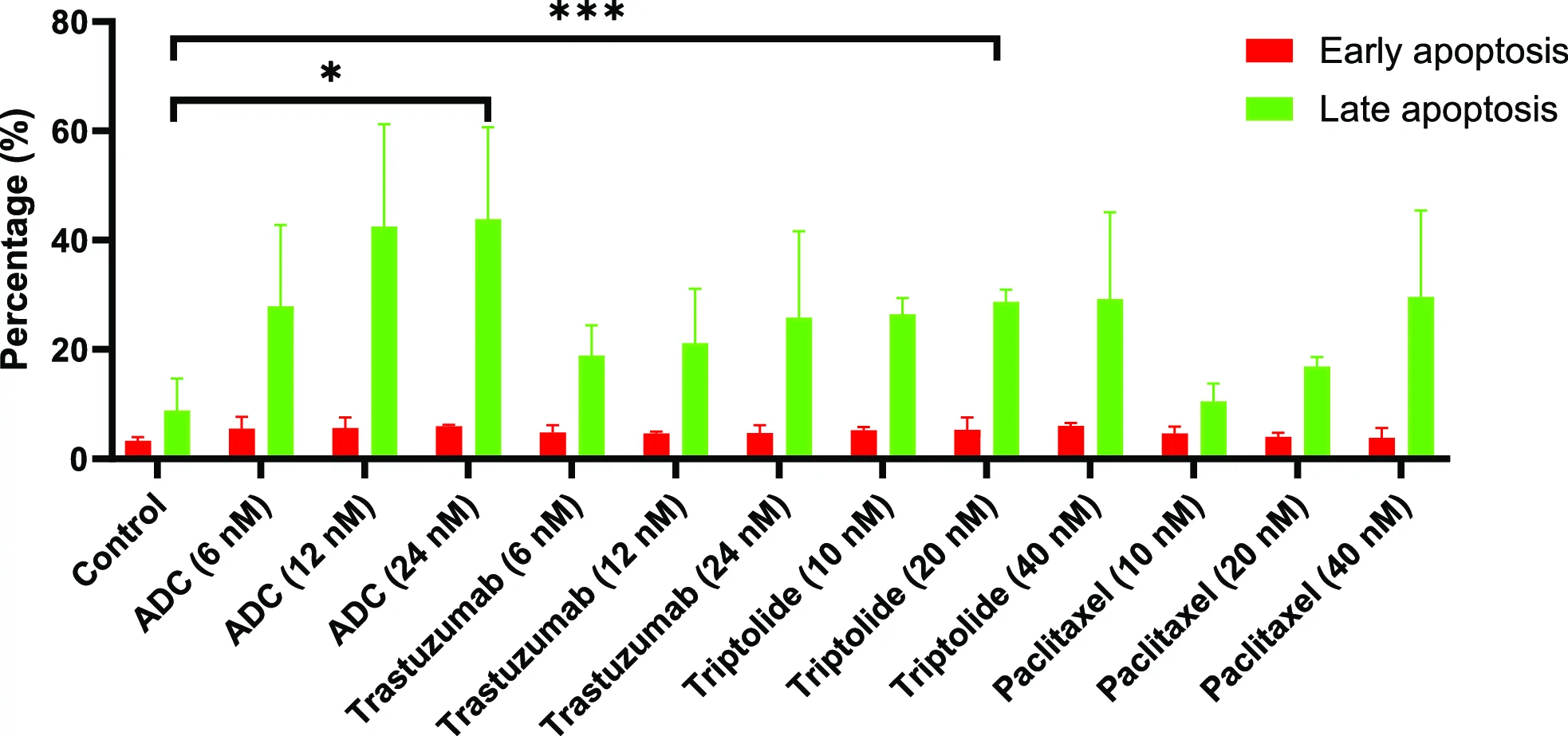
Percentage of late and early apoptosis for SKOV-3 after treatments of ADC, trastuzumab, triptolide, and paclitaxel. The significance levels between control and treatment of *p* < 0.05 and *p* < 0.001 are represented * and ***, respectively. e.g., *p* = 0.02 (control vs ADC, 24 nM); *p* = 0.0009 (control vs triptolide, 20 nM).

### 2.4 Cell Cycle Effects

To further characterize the effect of the drug, SKOV-3 cells were stained with PI and analyzed by flow cytometry (Figure 7 and Supporting Information). The cell cycle analysis shows that ADC increased the percentage of cells in the sub-G_1_ phase compared to the control. Triptolide and paclitaxel at lower concentrations also showed an increase in the population of cells in the subG_1_ phase but not as prominently as ADC. The cell counts in the G_0_-G_1_ phase decreased slightly, while cell counts at the S and G_2_-M phases increased with ADC. Trastuzumab did not show significant changes compared with the control. As expected, a decline in the population of cells in G_0_-G_1_ and an increase in G_2_/M phases were seen with paclitaxel.

**Figure 7:**
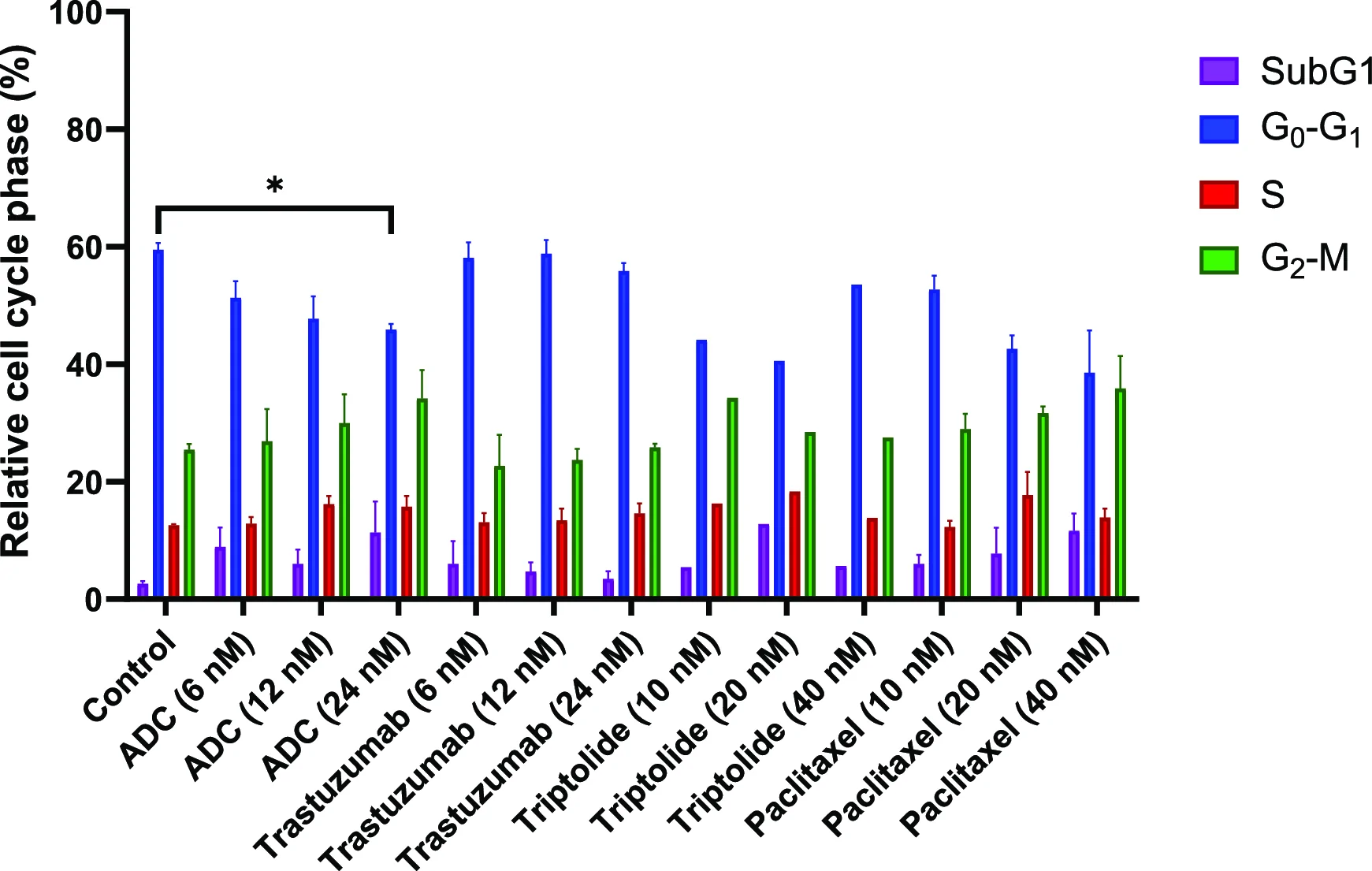
Relative cell cycle phase (%) for SKOV-3 after addition of ADC and trastuzumab at 6, 12, and 24 nM (A); triptolide and paclitaxel at concentrations 10, 20, and 40 nM (B). *p* = 0.034, control vs ADC (24 nM).

## 3 Discussion

ADCs constitute an expanding class of chemotherapeutic agents. The growing number of clinical trials and the recent approval of additional ADCs for clinical use^14^ have intensified interest in their continued development. Despite their promising cytotoxic activity, the pharmacological performance of conventional ADCs remains suboptimal. Previous approaches have utilized recombinant methods to overcome heterogeneity issues, and although notable progress has been achieved, these processes are incredibly complex, requiring extensive antibody engineering or conjugation site optimization.^12,18^

We have successfully synthesized a homogeneous ADC utilizing a triptolide payload and DBM linker chemistry. By employing interchain cysteine cross-linking across the native disulfide bridges, we achieved a controlled drug-to-antibody ratio (DAR) of 4. While previous triptolide-based ADCs were largely heterogeneous, only one prior homogeneous example has been documented using divinylsulfonamide (BVS) chemistry, which demonstrated efficacy in breast cancer models.^30^ In this study, we expand the therapeutic scope of the triptolide-based ADC by evaluating the *in vitro* cytotoxicity of this novel DBM-linked construct against the ovarian cancer cell lines.

The synthesized ADC exhibited exceptional *in vitro* cytotoxicity, maintaining a potency comparable to the free triptolide payload and outperforming the clinically standard chemotherapeutic paclitaxel. We observed differential potency between the two ovarian cancer cell lines: the ADC was more potent against SKOV-3 cells than OVCAR-8 cells, which correlates with the higher HER2 expression profiles previously reported^36^ for SKOV-3. The reduction of shed HER2 in the culture media by both trastuzumab and the ADC further suggests target-specific engagement. However, the relatively narrow margin between the IC_50_ values of the two cell lines (5.0 vs 9.0 nM) indicates that the selectivity of the ADC cannot be attributed exclusively to HER2-mediated internalization. This limited selectivity is heavily influenced by the intrinsic properties of the payload itself. Free triptolide demonstrated baseline differences in potency between the two cell lines, suggesting that inherent cellular susceptibility, potentially driven by variations in apoptotic priming, stress-response pathways, or reliance on survival signaling, is a critical determinant of cytotoxicity. Furthermore, the high potency and passive membrane permeability of triptolide raise the likelihood of target-independent intracellular entry, alongside a potential bystander killing effect following payload release.

This nuanced balance of efficacy and selectivity underscores the pivotal role of the DBM linker design. Structurally, the DBM linker attaches to the antibody through a stable thioether bond, which resists cleavage under physiological conditions and minimizes premature systemic deconjugation. The connection between the DBM core and the triptolide payload is formed through an ester bond, which is generally stable following ADC internalization within cancer cells.^37^ The cytotoxic payload, along with its linker, is likely released through lysosomal degradation of the ADC. Trastuzumab emtansine (Kadcyla), an FDA-approved ADC, incorporates an ester linkage connecting the maytansinoid payload DM1 to the SMCC linker, which is in turn, attached to the antibody via a thioether bond, closely resembling our design.^37^ Future work will focus on quantifying the release kinetics of the triptolide payload to better elucidate the mechanism of action of the ADC.

To elucidate the downstream cellular mechanisms driving this cytotoxicity, SKOV-3 cells were selected for functional assays due to their higher HER2 expression and sensitivity. The ADC demonstrated clear mechanistic superiority over parental trastuzumab by actively triggering programmed cell death, as evidenced by a significant upregulation of caspase-3/7 activity. This apoptotic phenotype was corroborated by flow cytometry, which revealed a distinct accumulation of SKOV-3 cells in the sub-G_1_ phase of the cell cycle. These findings align with the established molecular mechanism of triptolide, which covalently targets the XPB subunit of the TFIIH complex to inhibit RNA polymerase II–dependent transcription.^25^ This transcriptional arrest rapidly depletes short-lived pro-survival proteins such as Mcl-1, driving the cell efficiently toward apoptosis.^38^

A few limitations of the present study warrant acknowledgment. First, our evaluation was restricted to a single homogeneous ADC construct featuring a short, noncleavable linker as a proof-of-concept. Future iterations should explore a broader library of constructs, incorporating both cleavable and noncleavable linkers of varying lengths to optimize payload release kinetics. Second, while the therapeutic potential was demonstrated in a 2D *in vitro* setting, these models do not fully replicate the physiological complexity of the tumor microenvironment. Subsequent studies utilizing 3D organoid platforms^39^ or *in vivo* murine models are essential for rigorously assessing the efficacy, systemic toxicity, and pharmacokinetic profile of these conjugates.

## 4 Conclusion

This study successfully developed a homogeneous, trastuzumab- and triptolide-based ADC using a site-specific and stable linker designed to address the high mortality rates associated with ovarian cancer. The therapeutic potential of this ADC was supported by several key findings. The ADC demonstrated significant cytotoxic potency in the HER2-overexpressing cell line SKOV-3. In antiproliferative assays, the ADC exhibited superior potency compared to both the naked antibody (trastuzumab) and the positive control (paclitaxel). Its targeted nature was confirmed by a significant reduction in the level of HER2 following treatment, resulting in apoptosis. In summary, the developed ADC represents a promising targeted therapy for HER2-positive ovarian cancers by combining potent apoptotic induction with precise molecular targeting.

## 5 Methods

All materials and chemical reagents were purchased from Sigma–Aldrich UK and used as received. Trastuzumab (Herceptin) was purchased from Roche (Basel, Switzerland) and reconstituted according to the manufacturer’s instructions. ^1^H (400 MHz) and ^13^C NMR (100 MHz) spectra were recorded to confirm the identity of DBM in CD_3_OD and TP-DBM in CDCl_3_, respectively. Both compounds, DBM and TP-DBM were further analyzed using liquid chromatography-electrospray ionization mass spectrometry (LC/ESI-MS) and MALDI-MS, respectively. The ADC mass was analyzed using an Autoflex MALDI-TOF instrument in linear-only mode (Bruker). No unexpected or unusually high safety hazards were encountered in all the experiments.

### 5.1 Synthesis of 3,4-Dibromomaleimide-*N*-Acetic Acid, Dibromomaleimide (DBM) Linker

Glycine (120 mg, 1 mmol) was added to a solution of 3,4-dibromofuran-2,5-dione (256 mg, 1 mmol) in acetic acid (5 mL). The solution was stirred at room temperature for 10 min until all solids had dissolved. The mixture was then heated to 100 °C for 18 h. Thin-layer chromatography (TLC) was used to indicate the reaction had reached completion on precoated silica gel 60 F_254_ plates. Dichloromethane-methanol (5:1 v/v) was used as the mobile phase, and an iodine chamber was used to visualize spots. The reaction mixture was concentrated under vacuum. Toluene (10 mL) was added, and the solution again was concentrated under vacuum. The remaining solid was dissolved by dichloromethane: methanol (5:1 v/v). The subsequent solution was purified by silica gel chromatography (eluent: dichloromethane-methanol 0–10% with 1% acetic acid). The concentration of pure fractions afforded 164 mg (0.52 mmol, 44% yield) of dibromomaleimide derivative (3,4-dibromomaleimide-*N*-acetic acid). ESI-MS, *m*/*z*: 311.80 calcd. for [M + H]^+^: 311.85. ^1^H NMR and ^13^C NMR data (Supporting Information) are consistent with reported.^23^

### 5.2 Synthesis of Triptolide-Dibromomaleimide (TP-DBM) Payload

3,4-Dibromomaleimide-*N*-acetic acid (31 mg, 0.1 mmol) was added to acetonitrile (ACN) (2 mL). DMAP (5.5 mg) was added, along with triptolide (54 mg, 0.15 mmol). To this reaction mixture, DIC (15 mg, 0.12 mmol) was added, and the solution was stirred at 0 °C for 5 min and then at 20 °C for 3 h. TLC was conducted to ensure the reaction had reached completion (eluent: dichloromethane-methanol (20:1 v/v)), using UV light to visualize spots. Precipitated urea was filtered off, and the filtrate was concentrated under vacuum. The residue was taken up by ethyl acetate. The ethyl acetate solution was washed with 0.5 N HCl and dried over anhydrous sodium sulfate, and evaporated under reduced pressure.

The crude product was separated using high-performance liquid chromatography (HPLC). Agilent 1220 Infinity II LC system was used with a 5 μm particle size, 9.4 × 250 mm column (Phenomenex, UK; Jupiter C18 reverse phase, 300 Å). For analysis, the mobile phase used consisted of (solvent A) Trifluoroacetic acid (TFA) 0.1% in water and (solvent B) 80% acetonitrile with water and 0.1% TFA (80:20). Gradient HPLC method was used with a total run time of 25 min. The UV detector wavelength was 215 nm and flow rate was maintained at 1 mL/min. The initial mobile phase composition was 20% for solvent B, changed linearly to 80% over 20 min followed by a change to 100% held for 5 min. Separation was conducted on the same system with a semipreparative HPLC column (Phenomenex, UK; Jupiter C18 reverse phase, 300 A) of 5 μm particle size, 9.4 × 250 mm, using gradient HPLC method. UV detector wavelength was set at 215 nm, and flow rate was maintained at 4 mL/min. Initial mobile phase composition was 30% for solvent B, changed linearly to 100% over 16 min and held at 100% for 4 min. Purification afforded triptolide DBM (TP-DBM, 14 mg, 0.021 mmol, 17% yield). ESI-MS, *m*/*z* 653.9483, calcd. for [M + H]^+^: 653.9974. ^1^H NMR and ^13^C NMR spectroscopic data are shown in Table 1 and Supporting Information.

### 5.3 Synthesis of Antibody-Drug Conjugate

The conjugation procedure was adapted from a previous method with minor modifications.^23^ The procedure was performed in ammonium bicarbonate buffer (pH 8.5). Briefly, trastuzumab (17.5 mg, equivalent to 35 mg of Herceptin formulation, 0.12 μmol) was diluted in 2.5 mL of buffer solution and run through a PD-10 gel filtration column using 3.5 mL buffer. 96 μL of TCEP (10 mM, 0.96 μmol, 8 equiv) was added to trastuzumab solution. The reaction was mildly agitated and incubated at 37 °C for 2 h. TP-DBM payload (0.6 μmol, 5 equiv) in anhydrous DMF (60 μL, 10 mM) was added and the reaction mixture was further incubated at 20 °C for 60 min. Finally, extra reagents were removed by ultrafiltration using the PD-10 column (*M*_r_ exclusion limit 5000) with 50 mM phosphate buffer, pH 7.5. The concentration of trastuzumab was quantified using the extinction coefficient^40^ of trastuzumab at 280 nm, 225000 M^–1^cm^–1^. The ADC was also quantified using this coefficient, as the hydrolyzed linker and triptolide have no absorption at 280 nm.

### 5.4 Cell Culture

Human ovarian cancer cell lines, SKOV-3 and OVCAR-8 (ATCC), were cultured in RPMI 1640 (Lonza) supplemented with 10% fetal bovine serum (FBS, Lonza), 5000 μg/mL penicillin–streptomycin (Lonza), and 2 mM glutamine (Lonza). Cells were maintained in a humidified incubator at 37 °C with 5% CO_2_.

### 5.5 Cytotoxicity Assay

Cell growth was assessed using the colorimetric WST-8 kit (PromoCell). Cells were seeded in 96-well plates at optimized densities of 2,000 cells/well for the fast-growing OVCAR-8 cells and 5,000 cells/well for the slow-growing SKOV-3 cells in 80 μL of medium. Following attachment, cells were treated with 2-fold serial dilutions of ADC, triptolide, TP-DBM, or paclitaxel. After a 72-h incubation, 10 μL of WST-8 reagent was added to each well, followed by a 2-h incubation at 37 °C. Absorbance was measured at 460 nm using a multimode microplate reader (Synergy 2, BioTEK). Dose–response curves and IC_50_ values (*n* = 3) were generated via nonlinear regression using a four-parameter logistic model (GraphPad Prism 9.0).

### 5.6 HER2 Enzyme-Linked Immunosorbent Assay (ELISA)

Extracellular HER2 (ErbB2) expression was quantified by using a human ErbB2 ELISA kit (Abcam). A 50 ng/mL ErbB2 stock was serially diluted in 1x assay diluent B to create an 8-point standard curve, with the final tube serving as a zero-concentration control. Detection antibody and HRP-conjugated streptavidin concentrates were diluted in 1x assay diluent B to their working concentrations. Samples (cell culture supernatants from untreated, and OVCAR-8, trastuzumab and ADC-treated SKOV-3 at 6 and 12 nM) and standards were incubated in a precoated plate. Following a wash cycle using 1x wash buffer, biotinylated antihuman ErbB2 antibody was added, followed by HRP-streptavidin. The signal was developed using the TMB substrate, neutralized with stop solution, and quantified by measuring absorbance at 450 nm. HER2 present in medium through the shedding of its extracellular domain^35^ was determined by interpolation from the standard curve (*n* = 3).

### 5.7 Apoptosis Caspase-3/7 Assay

Apoptosis induction was measured using the Caspase-Glo 3/7 kit (Promega). SKOV-3 cells were seeded at 5,000 cells/well in 96-well plates. After 24 h, cells were treated with ADC or trastuzumab (6, 12, and 24 nM), or triptolide or paclitaxel (10, 20, and 40 nM). Following a 48-h incubation, 25 μL of Caspase-Glo reagent was added to each well. Plates were agitated on a microplate shaker in the dark for 40 min prior to luminescence measurement at 460 nm (BioTEK Synergy 2, USA). Results were normalized to parallel cytotoxicity data obtained via the WST-8 assay. The normalized caspase data (*n* = 3) were statistically analyzed on GraphPad Prism 9.0.

### 5.8 Cell Morphology Assay

SKOV-3 cells were seeded at 10 000 cells/well in 500 μL of medium into 24-well plates. At 24 h post-seeding, cells were treated with ADC, trastuzumab, triptolide, or paclitaxel at the concentrations described above. Following 48- and 72-h incubations, morphological changes were captured at 20× magnification using an Olympus CKX41 light microscope.

### 5.9 Flow Cytometry

SKOV-3 cells were seeded at 20,000 cells/well in 24-well plates and treated with ADC for 48 h. ADC and trastuzumab (6, 12, and 24 nM) were added 24 h after seeding alongside triptolide and paclitaxel (10, 20, and 40 nM). Supernatants were collected into 15 mL polypropylene tubes. Adherent cells were detached with 500 μL trypsin (37 °C for 7–10 min), neutralized with 500 μL media, and pooled with their respective supernatants. Cell suspensions were then centrifuged at 150 x *g* for 3 min. Pellets were transferred to 2 mL tubes, centrifuged at 300× *g* for 5 min at 4 °C, and washed with 1 mL phosphate-buffered saline (PBS). Final pellets were processed for Annexin V (to detect phosphatidylserine externalization) (MACS, Bergisch Gladbach, Germany) and propidium Iodide (PI) (to detect compromised membrane integrity) staining to distinguish between live, early apoptotic, late apoptotic, and necrotic populations. All data were collected using the CytExpert software, and dot plots were generated using the phycoerythrin (PE) and fluorescein isothiocyanate (FITC) channels. For every drug concentration used, 3 replicates were obtained, and after which data analysis was performed on GraphPad Prism 9.0 to determine statistical differences in % late and early apoptosis between treatments and the control, and within different concentrations of the same treatment.

### 5.10 Cell Cycle Assay

Cell cycle distribution was analyzed using PI and ribonuclease (RNase) staining to quantify DNA content. SKOV-3 cells were seeded at 20,000 cells/mL in 24-well plates and treated with ADC, trastuzumab, triptolide, or paclitaxel at the concentrations previously described. Following a 48-h incubation, cells were harvested, washed in 1 mL PBS, and fixed in 1 mL of 70% ethanol for 45–60 min. Fixed cells were centrifuged (500× *g*, 5 min), washed twice with PBS, and incubated with 300 μL PI and 50 μL RNase solution for 30–45 min in the dark at room temperature. Analysis was performed on a Beckman Coulter CytoFlex flow cytometer. Forward scatter (FSC) and side scatter (SSC) gating were utilized to exclude cell debris and aggregates. Histograms representing the G_0_/G_1_, S, and G_2_/M phases were generated using CytExpert software.

### 5.11 Statistical Analysis

All three independent repeat data, once collected, were first sorted out on Microsoft Excel, followed by analysis using GraphPad Prism 9.0, whereby both statistical analysis and graphical representations were obtained. Data are presented as the mean with the standard deviations (SD), except for IC_50_ as standard error of mean (SEM) (*n* = 3). One-way and two-way ANOVA were made depending on the number of independent variables being investigated, with the former being for one and the latter being for two independent variables.

## Supplementary Material



## Notes

HPLC traces of TP-DBM payload, trastuzumab, and ADC; ^1^H and ^13^C NMR spectra for DBM linker and TP-DBM payload; SEC chromatographs of trastuzumab and ADC; representative samples from cell cycle analysis and from Annexin V and propidium iodide (PI) staining of SKOV-3 using flow cytometry; additional experimental details and methods (PDF)
