# Supplementary material for: Development of a Homogeneous Trastuzumab-Triptolide Conjugate for Targeted Therapy of HER2-Overexpressing Ovarian Cancer
Source: Bioconjug Chem. 2026 Aug 21;37(9):1824–32. doi: 10.1021/acs.bioconjchem.6c00171 (PMC13587595; doi:10.1021/acs.bioconjchem.6c00171)
Supplement: Supplementary file 1 [file bc6c00171_si_001.pdf]

Supporting Information

Development of a Homogeneous Trastuzumab-Triptolide Conjugate for Targeted Therapy of HER2-Overexpressing Ovarian Cancer

Idowu E. Fadayomi[1], Maria Jangan[2], Dilna Varghese[1], Elisabete Pires[3], James McCullagh[3], Alan Richardson[2], Adrian Murray Brunt[4], and Wen-Wu Li[1]*

[1]School of Life Sciences, Keele University, Staffordshire, ST4 7QB, UK

[2]School of Health Allied Professional and Pharmacy, Keele University, Staffordshire, ST5 5BG, UK

[3]Department of Chemistry, University of Oxford, Oxford, OX1 3TA, UK

[4]School of Medicine, Keele University, Staffordshire, ST5 5BG, UK

A.

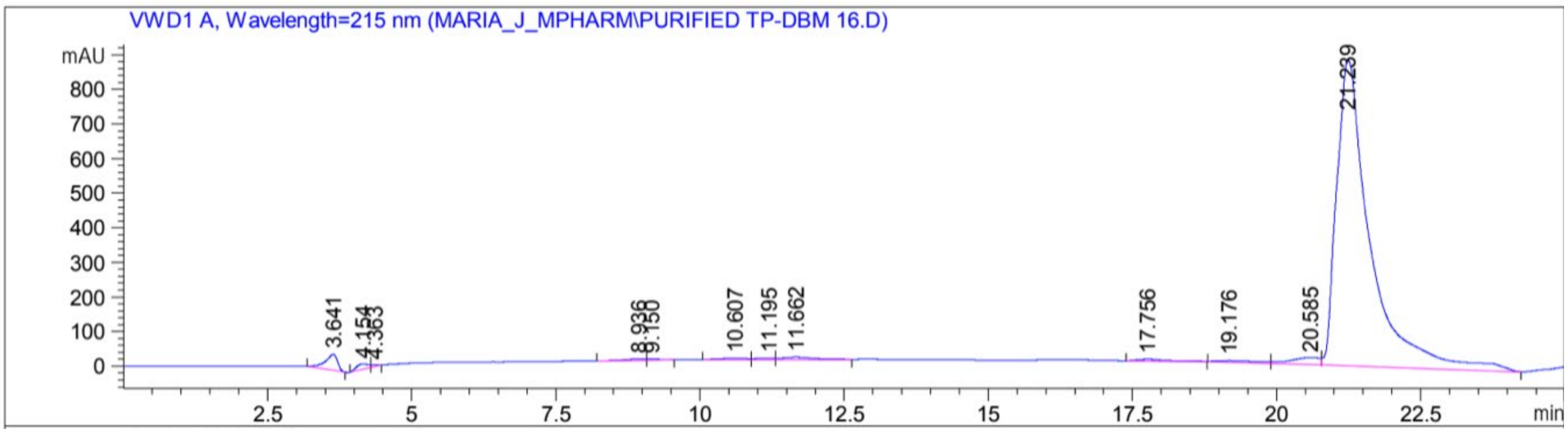

B.

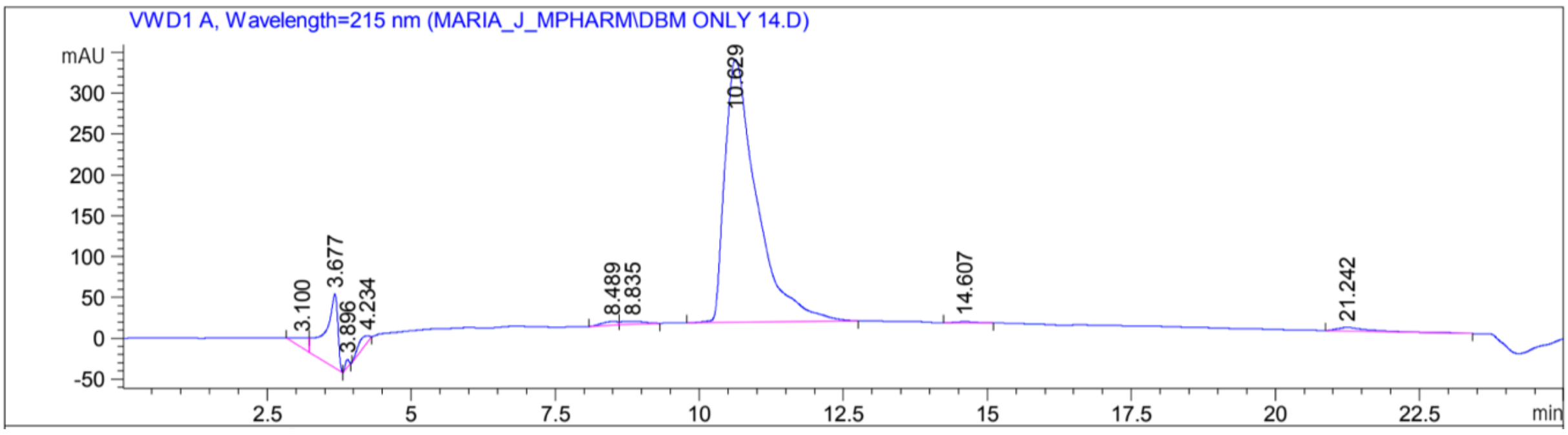

C.

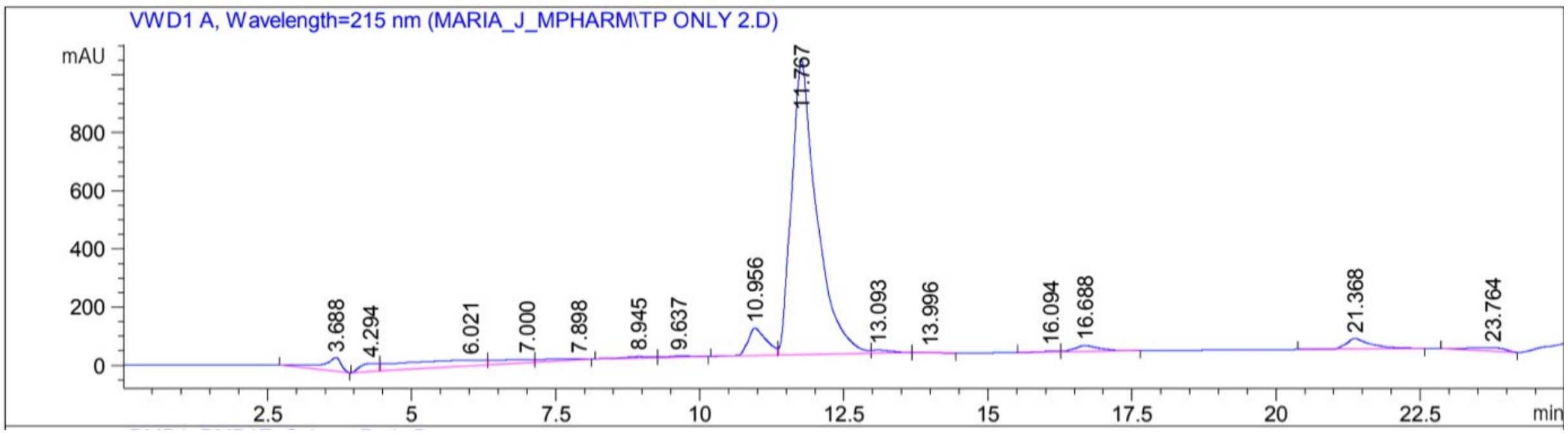

Figure S1: HPLC of TP-DBM payload (A), Triptolide (B) and DBM linker (C)

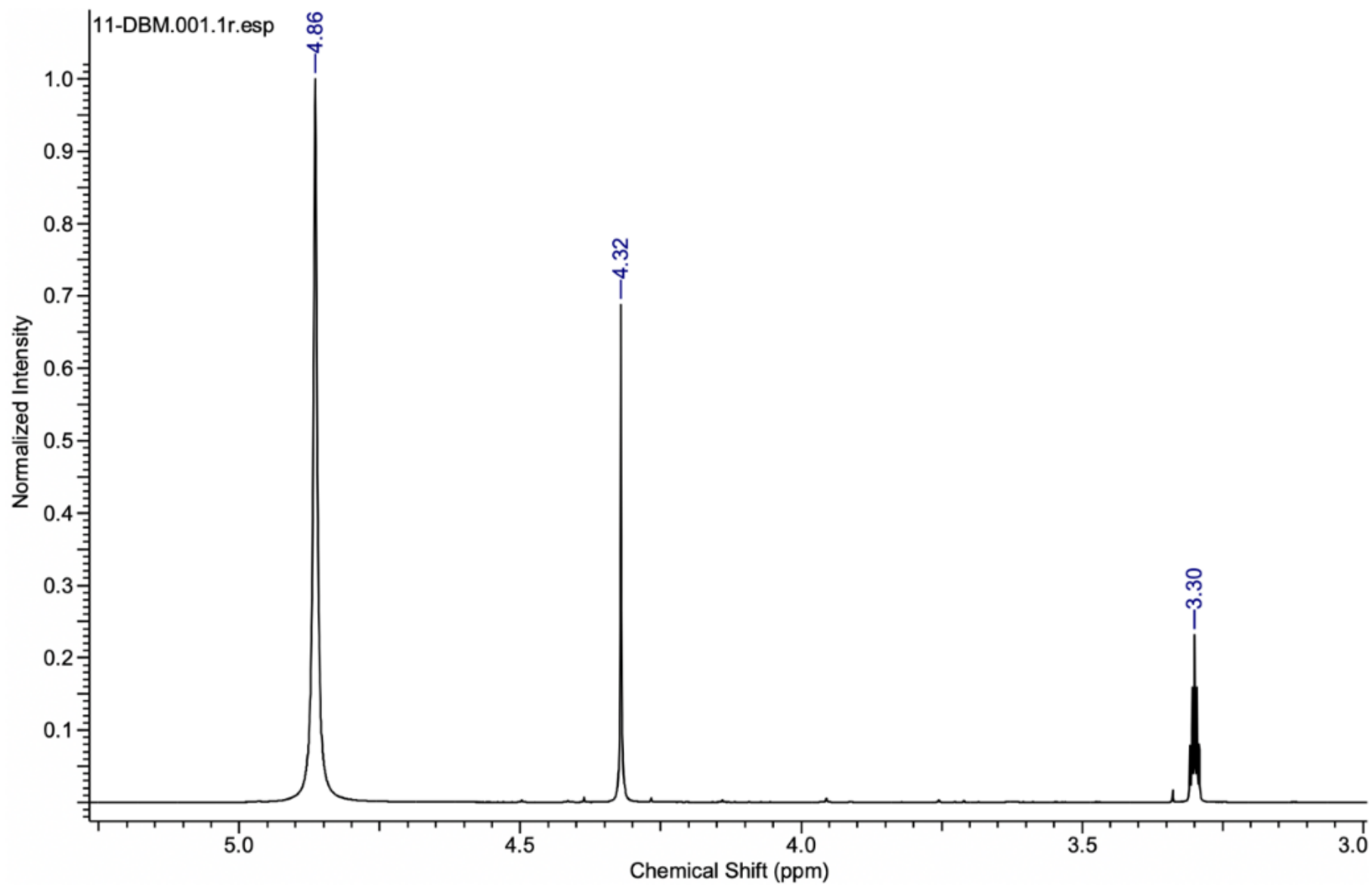

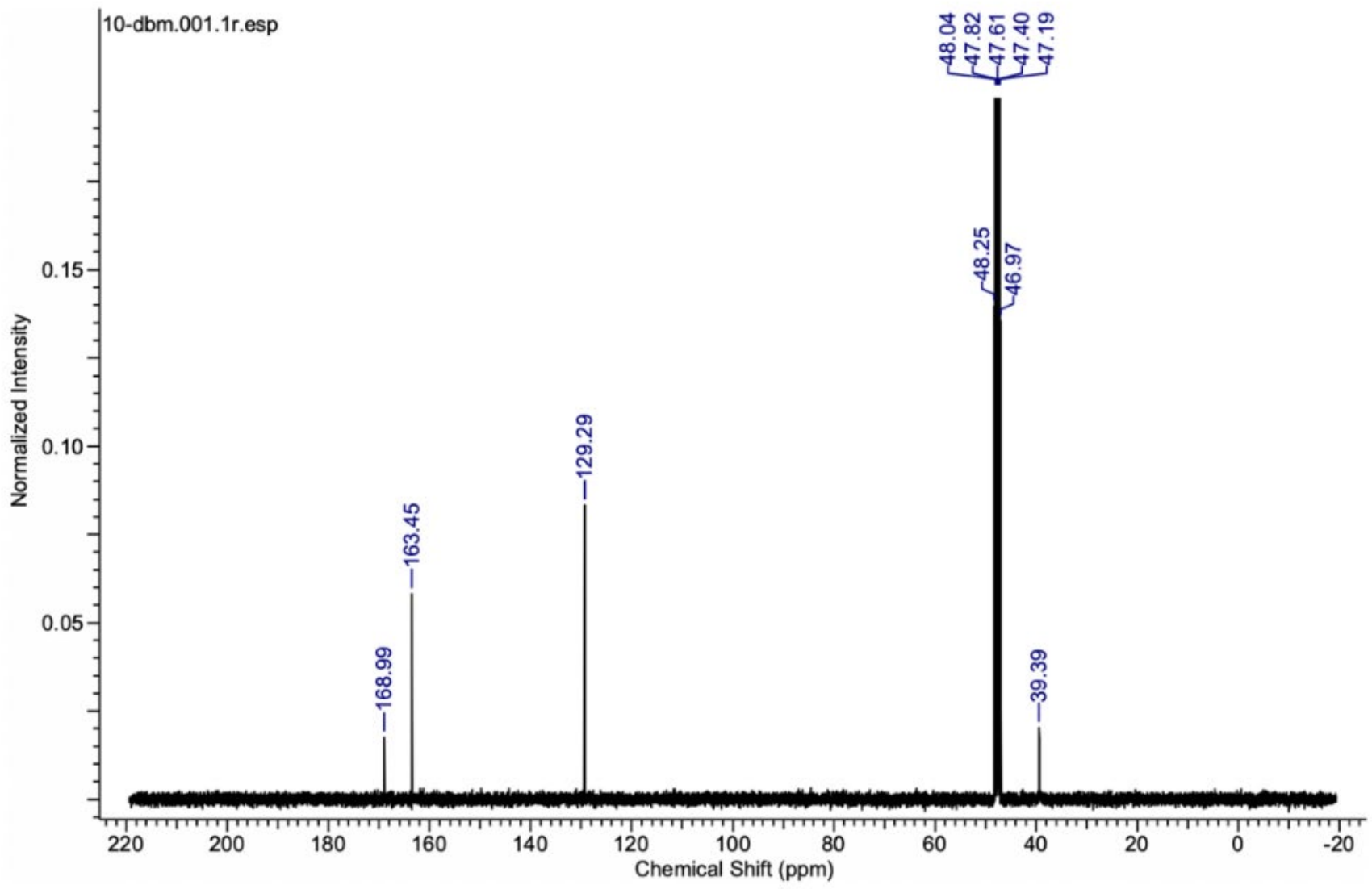

Figure S2: ^{1}H and ^{13}C NMR spectra of dibromomaleimide (DBM) linker in $CDCL_3$ respectively.

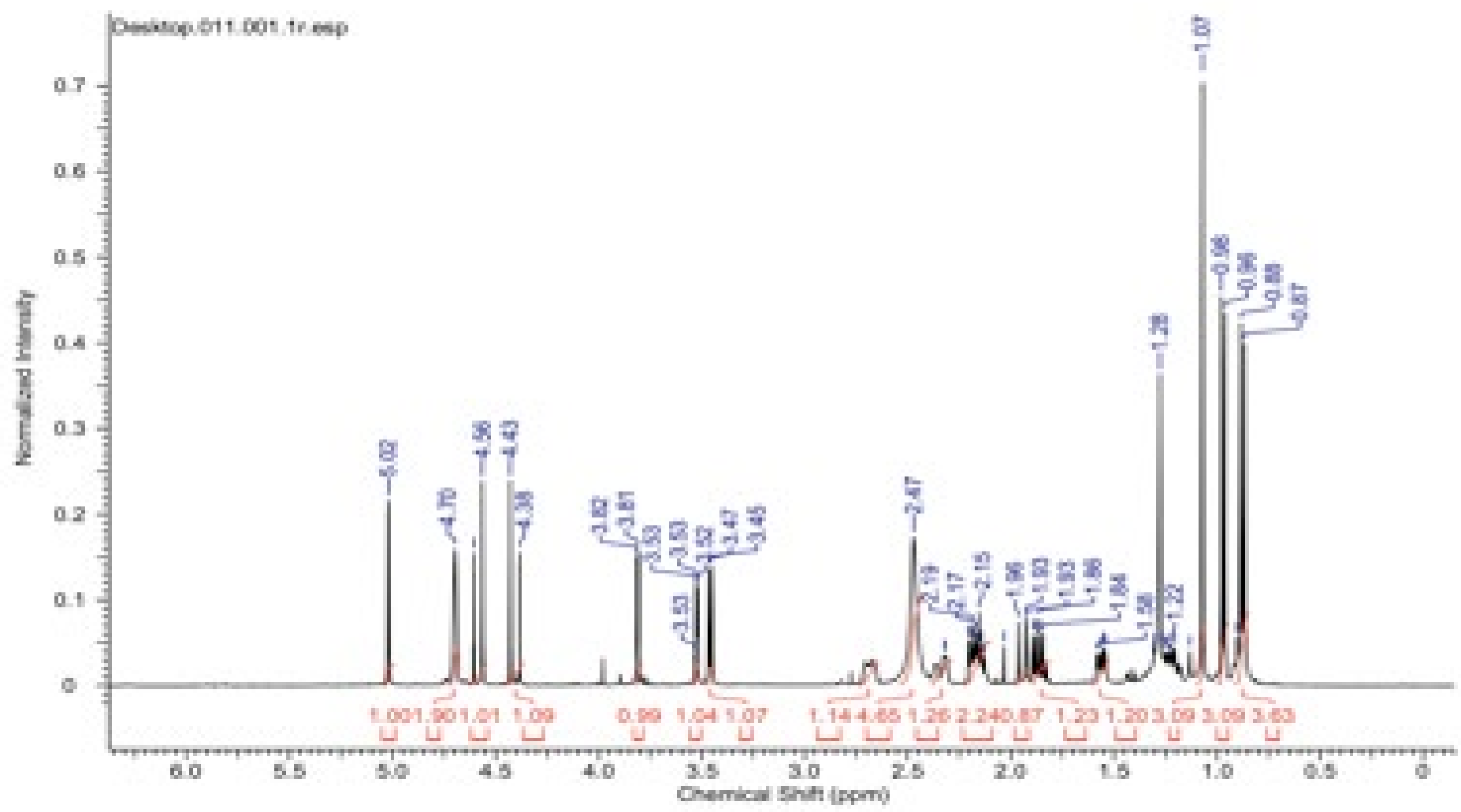

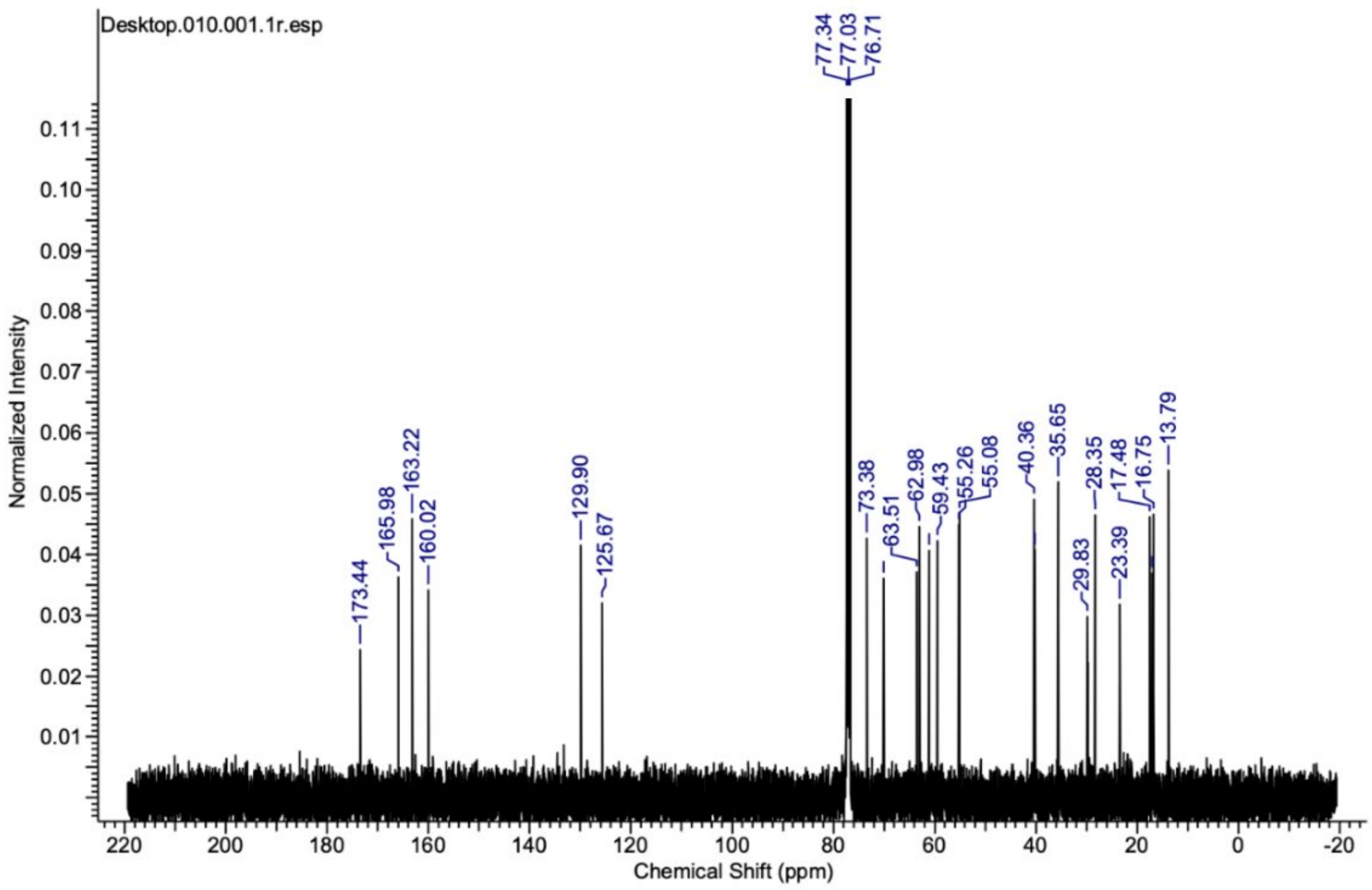

Figure S3. ^{1}H NMR (top) and ^{13}C NMR (bottom) spectra of the TP-DBM derivative in CD_3OD respectively.

.

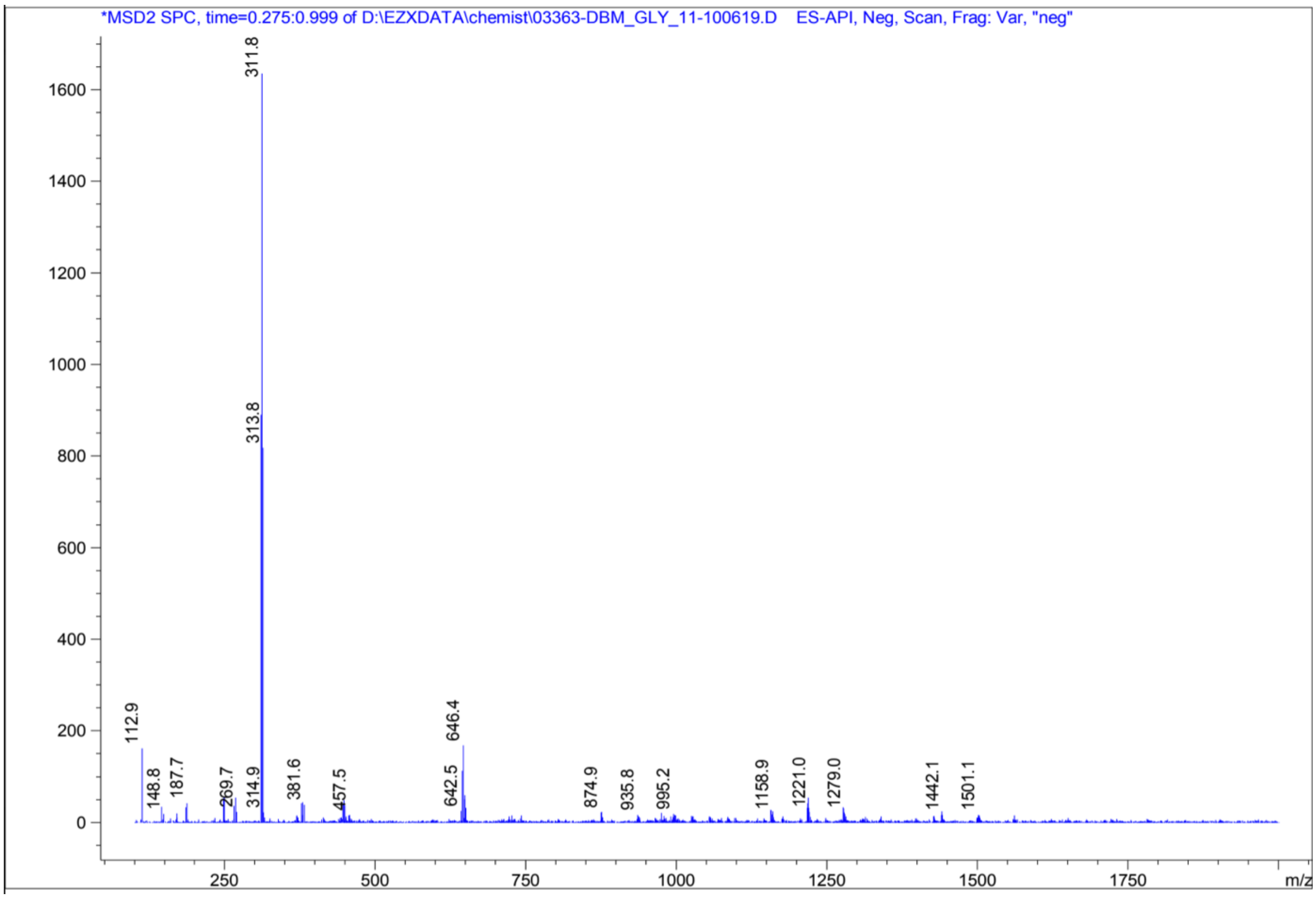

Figure S4 : LC/ESI-MS spectrum of DBM linker. The spectrum shows a peak at m/z 311.8 $[M + H]^+$ consistent with the calculated exact mass of DBM.

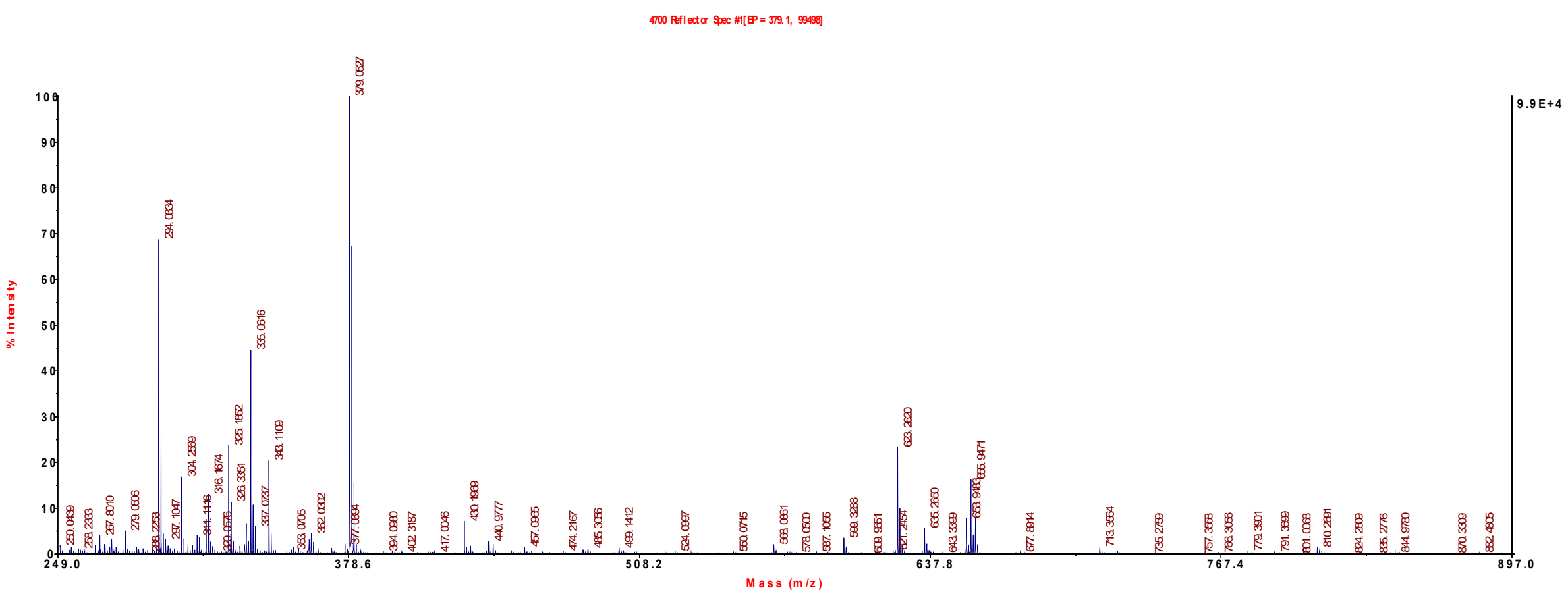

Figure S5: MALDI MS spectrum of TP-DBM. MALDI MS data for TP-DBM is also consistent with the calculated exact mass as a peak can be observed at m/z 653.9483 $[M+H]^+$.

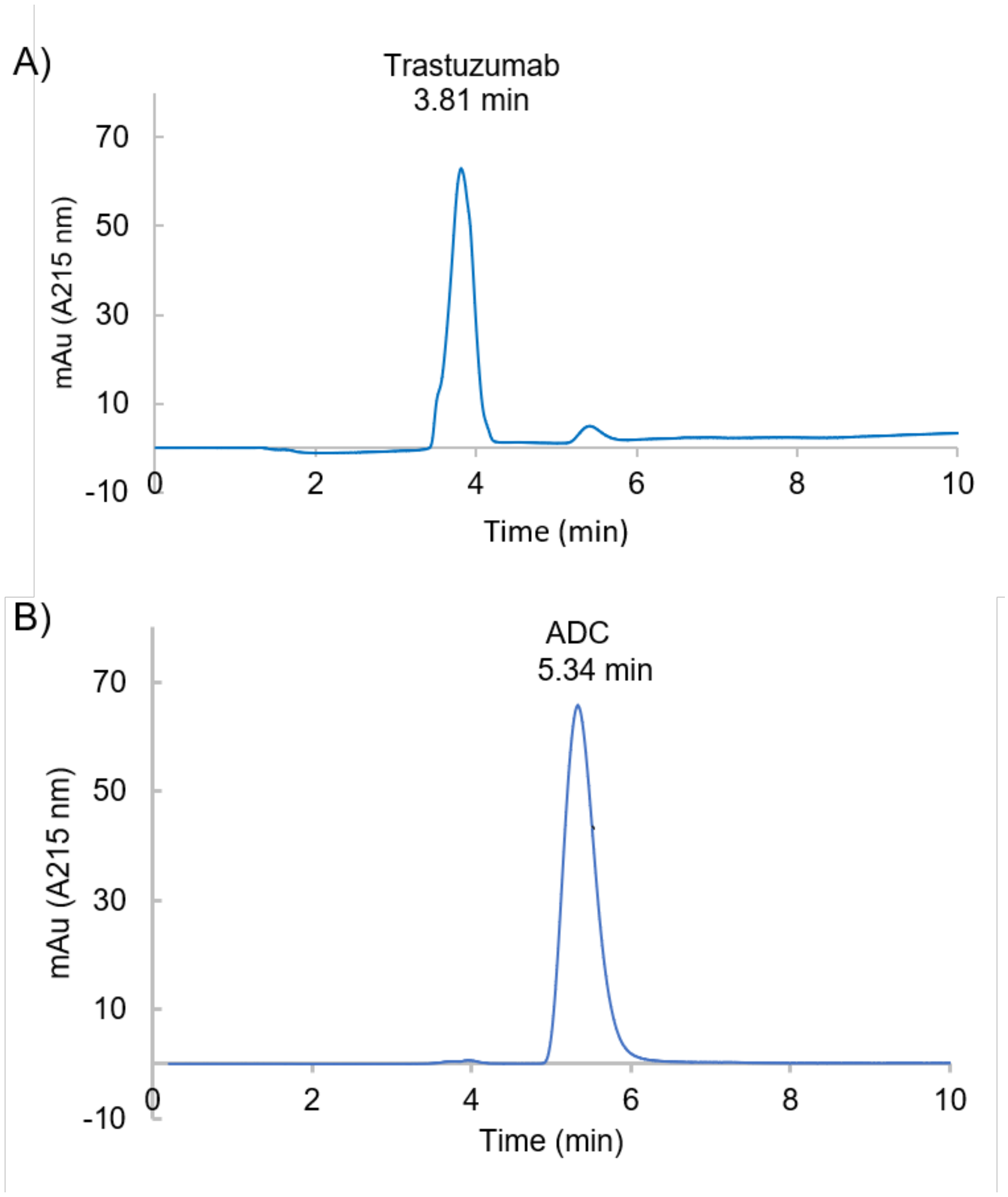

Figure S6: HPLC of unmodified trastuzumab (A) and trastuzumab-triptolide conjugate (ADC) (B). The retention time of ADC is increased by 1.53 min due to the covalent binding of hydrophobic triptolide. A single, symmetric ADC peak indicates a homogeneous population with a consistent antibody–drug ratio (ADR).

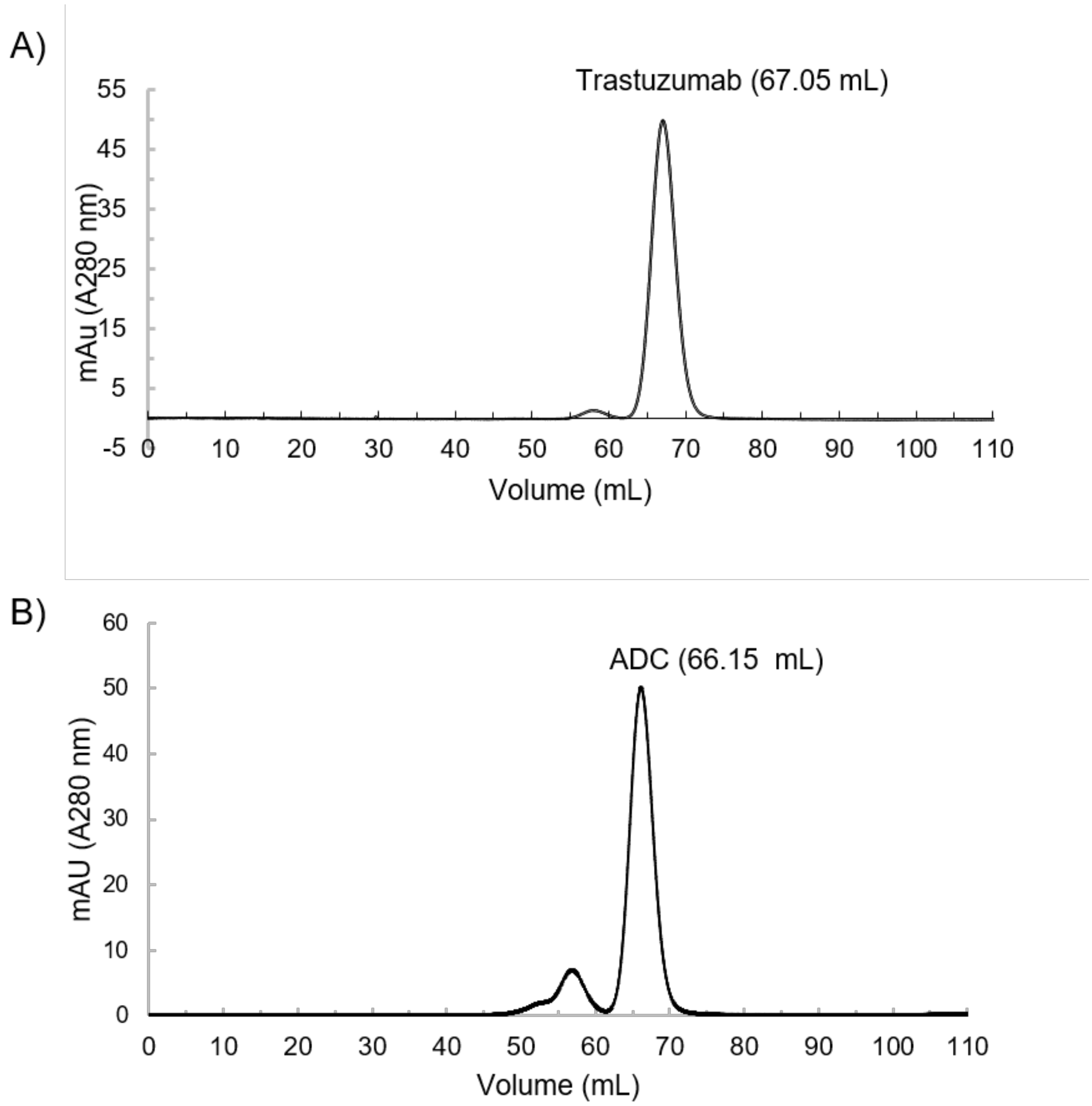

Figure S7: Size exclusion chromatography (SEC) of unmodified trastuzumab (A) and trastuzumab-triptolide conjugate (ADC) (B). The retention time of ADC is decreased by 0.9 min compared to that of trastuzumab due to the increase of its size because of covalent binding of four molecules of triptolide. ADC is predominantly monomeric (87%), with a minor fraction present as dimer (13%).

Figure S8: Representative samples from cell cycle analysis of SKOV-3 using flow cytometry, whereby the % cells in each cell cycle phase (SubG_1, G_0-G_1, S, G_2-M) is shown after ADC and trastuzumab (at 6, 12 and 24 nM); triptolide and paclitaxel (at 10, 20 and 40 nM) addition.

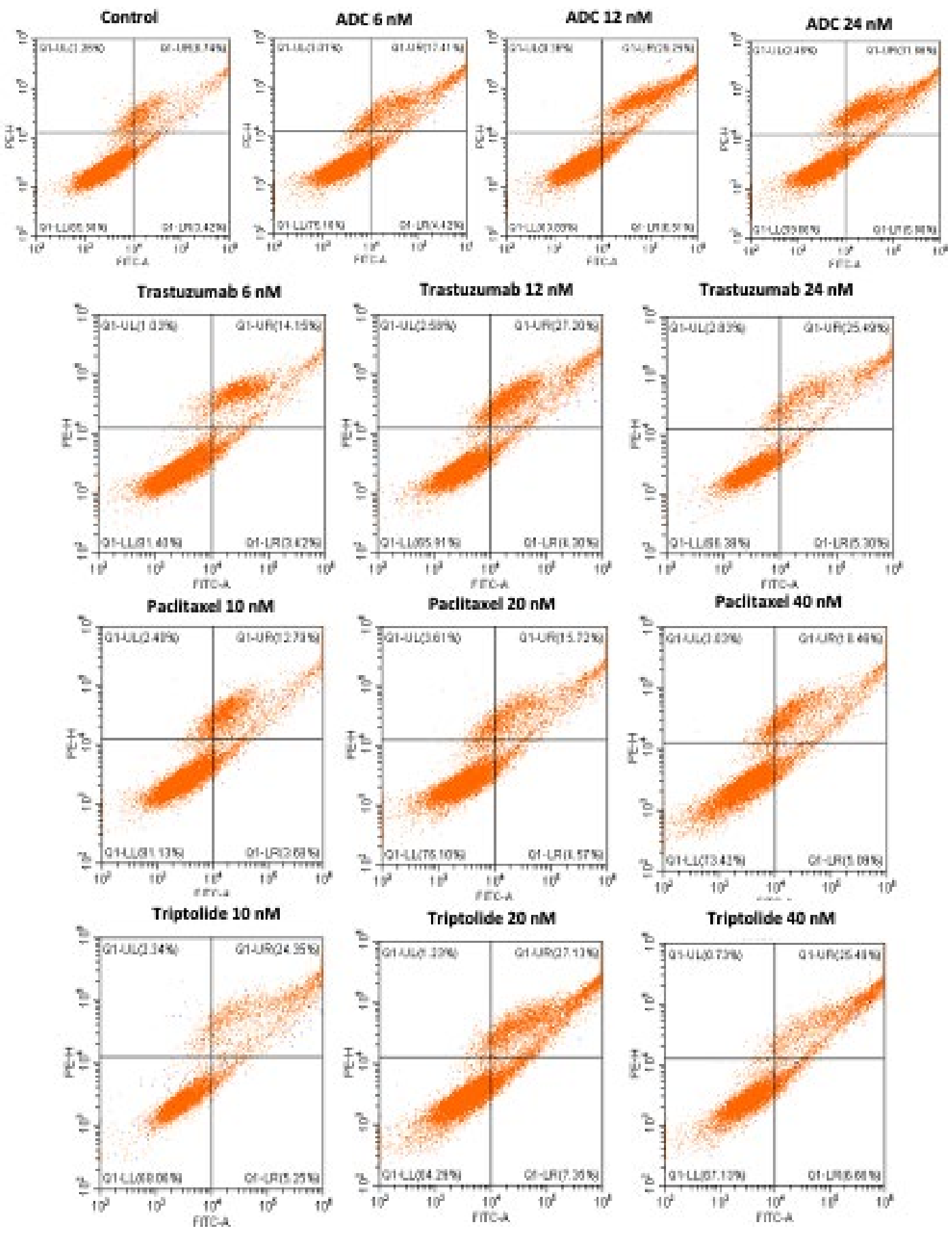

Figure S9: Representative plots of control, ADC and trastuzumab (6, 12, and 24 nM), paclitaxel and triptolide (10, 20 and 40 nM) from Annexin V and propidium iodide (PI) staining of SKOV-3 using flow cytometry. UR stands for upper right and represents % cells in late apoptosis. LR stands for lower right and represents % cells in early apoptosis. UL stands for upper left and represents % necrotic cells. LL stands for upper left and represents % live cells.

Size Exclusion Chromatography (SEC) Analysis of ADC Using ÄKTA System

The SEC analysis of antibody–drug conjugate (ADC) and trastuzumab was performed using an ÄKTA chromatography system. Separation was achieved on a HiLoad™ 16/600 column packed with Superdex™ 200 pg resin, which is suitable for resolving monoclonal antibodies and their aggregates.The column was equilibrated with phosphate-buffered saline (PBS) containing 20 mM sodium phosphate and 137 mM NaCl (pH 7.4) and operated at a flow rate of 1.0 mL/min under isocratic conditions. Detection of eluted proteins was carried out by

monitoring UV absorbance at 280 nm. For analysis, 0.5 mL of 1 mg/mL solutions of trastuzumab and ADC were injected separately onto the column. Elution profiles were recorded, and peaks corresponding to monomeric species and potential aggregates were evaluated based on retention time and peak area.

MALDI-TOF-MS

Trastuzumab and ADC samples after mixing with 2,6-Dihydroxyacetophenone (DHAP, 50 mM) in 2% TFA were analysed by MALDI Autoflex Speed (Bruker Daltonics, Bremen, Germany) equipped with a Nd: YAG @ 355 nm laser with a Variable repetition rate up to 2 kHz. The instrument parameters were used with the following settings: mass range: 30,000 to 210,000 Da, sample rate: 0,16 GS/s, laser shots: 1000, laser power: 100%, laser frequency: 100, and detector gain: 25kV. Spectra were obtained in positive ion mode. IgG (150,000 Da) was used as an external standard for mass assignment. FlexAnalysis software was used for processing the spectra acquired with the Bruker time-of-flight mass spectrometer.
